# The Prediction of ADL and IADL Disability Using Six Physical Indicators of Frailty: A Longitudinal Study in the Netherlands

**DOI:** 10.1155/2014/358137

**Published:** 2014-03-24

**Authors:** Robbert J. J. Gobbens, Marcel A. L. M. van Assen

**Affiliations:** ^1^Research & Development Centre Innovations in Care, Rotterdam University of Applied Sciences, Rochussenstraat 198, 3015 EK Rotterdam, The Netherlands; ^2^Department of Methodology and Statistics, Tilburg School of Social and Behavioral Sciences, Tilburg University, Tilburg, The Netherlands

## Abstract

Frailty is a predictor of disability. A proper understanding of the contribution of individual indicators of frailty in the prediction of disability is a requisite for preventive interventions. The aim of this study was to determine the predictive power of the individual physical frailty indicators: gait speed, physical activity, hand grip strength, Body Mass Index (BMI), fatigue, and balance, for ADL and IADL disability. The sample consisted of 505 community-dwelling persons (≥75 years, response rate 35.1%). Respondents first participated between November 2007 and June 2008, and a subset of all respondents participated again one year later (*N* = 264, 52.3% response rate). ADL and IADL disability were assessed by the Groningen Activity Restriction Scale. BMI was assessed by self-report, and the other physical frailty indicators were assessed with the TUG test (gait speed), the LAPAQ (physical activity), a hand grip strength test, the SFQ (fatigue), and the Four-test balance scale. All six physical frailty indicators were associated with ADL and IADL disability. After controlling for previous disability, sociodemographic characteristics, self-perceived lifestyle, and chronic diseases, only gait speed was predictive of both ADL and IADL disability, whereas there was a small effect of fatigue on IADL disability. Hence, these physical frailty indicators should be included in frailty assessment when predicting future disability.

## 1. Introduction

The number of disabled older persons is expected to increase worldwide during the coming decades [1]. Prevalence figures range from 30% for persons aged 75 and older to 40% for those persons aged 85 and older [2]. Disability is commonly defined as experiencing difficulty in carrying out activities that are essential to independent living—difficulties in performing activities of daily living (ADL) and/or instrumental activities of daily living (IADL) [3]. ADL functions are essential for an individual's self-care (e.g., dressing and feeding yourself), whereas IADL functions are more concerned with self-reliant functioning in a given environment (e.g., making the beds and shopping). Disability is associated with increased healthcare utilization and related costs [4], premature death [5–7], and impaired quality of life of the older population [8]. Hence, disability prevention in frail older persons is seen as a priority for research in geriatrics [9] and an important public health concern [10]. As a first step to disability prevention, this paper centers on the prediction of ADL and IADL disability, with the focus on the predictive power of frailty.

Frailty is a well-known predictor of disability [11, 12]. It is considered as a predisability state by the European, Canadian, and American Geriatric Advisory Panel (GAP) [13]. It is defined as a dynamic state affecting an individual who experiences losses in one or more domains of human functioning (physical, psychological, or social), which is caused by the influence of a range of variables and which increases the risk of adverse outcomes [14]. Previous studies have shown that of the three frailty domains (physical, psychological, and social), physical frailty was by far the most powerful predictor of disability [15, 16]. The strong association between physical frailty and disability is confirmed by studies in the United States [11], Canada [17], and the Netherlands [18], using different age groups (≥65 years [11], ≥75 years [17], and 75 to 80 years) [18]. Because of its strong association with physical frailty, we focus on the prediction of disability using physical frailty indicators.

A widely cited definition of physical frailty regards frailty as a biological syndrome of decreased reserve and resistance to stressors, resulting from cumulative decline across multiple physiologic systems, causing vulnerability to adverse outcomes [11]. Physical frailty encompasses a number of indicators. Well-established indicators of physical frailty are gait speed [19, 20], physical activity [21, 22], weight loss [23, 24], hand grip strength [25, 26], and balance [27]. Recently, a systematic review has concluded that each of aforementioned physical frailty indicators predicts future ADL disability [28]. This review showed that slow gait speed and low physical activity were the most powerful predictors. Another review, which used a broader definition of disability that also included IADL disability, found associations with gait speed, hand grip strength, upper and lower extremity function, and physical activity [29]. Four of the physical frailty indicators (gait speed, physical activity, weight loss, and hand grip strength), together with endurance, belong to the phenotype of frailty [11]. Nowadays the phenotype of frailty is a widely used operational definition of frailty. The phenotype was predictive of decline in ADL ability in 3- and 7-year follow-up in community-dwelling older people [11].

A proper understanding of the contribution of individual indicators of physical frailty in the prediction of disability is a requisite for preventive interventions. If individual indicators can predict disability this could be clinically useful in identifying older persons who might benefit from an intervention aimed at preventing disability. The aim of this study was to determine the predictive power of the individual physical frailty indicators: gait speed, physical activity, hand grip strength, Body Mass Index (BMI), fatigue, and balance, for disability. The first three indicators refer to the phenotype of frailty [11]; we added BMI, fatigue, and balance because these are also frequently used in studies of frail older persons; for example, the BMI was used in the Longitudinal Aging Amsterdam (LASA) [30] and in the Hispanic Established Populations for Epidemiologic Studies of the Elderly (EPESE) [31]; fatigue is one of the domains of the “FRAIL” scale, developed by a GAP [13], and two systematic reviews have shown that balance is a predictor of ADL disability in community-dwelling older people [28, 29]. We investigated the prediction of ADL, IADL, and disability (= ADL + IADL) by six physical frailty indicators, after controlling for the effects of background characteristics and previous disability of community-dwelling older people aged 75 years and older, one year later.

Previous studies also predicted disability using frailty indicators [28, 29]. Our study distinguishes itself from other studies in several ways. Most previous studies focused on either ADL [11, 27, 32] or IADL disability [33, 34], whereas we focus on ADL, IADL, and (total) disability. Moreover, we use a relatively short follow-up period of one year and we also use another set of frailty indicators.

## 2. Methods

### 2.1. Study Population and Data Collection

In 2007 a sample of 1,438 community-dwelling individuals aged 75 years and older was drawn randomly from a register of the municipality in Roosendaal (the Netherlands), a town of 78,000 inhabitants. A total of 505 persons participated in the study (35.1% response rate). Participants' physical frailty was assessed by several questions and measurement instruments. The participants were first assessed by trained interviewers between November 2007 and June 2008. Interview, physical measurement procedures, and interviewers' attitude were standardized through interviewers' participation in an eight-hour training course. The interviewers conducted personal face-to-face interviews using structured questionnaires, regarding sociodemographic characteristics, lifestyle, chronic diseases, and disability at the subject's residence. In addition, psychological and social frailty indicators were assessed. Completing the interview and physical measures took on average 75 minutes [35]. A subset of all 505 respondents of the sample was again interviewed and physical measures were taken one year later, between November 2008 and June 2009 (*N* = 264, 52.3% response rate).

The review board of the Faculty of Social and Behavioral Sciences at Tilburg University approved the study, and informed consent for the collection and use of information was obtained from all respondents.

### 2.2. Measures

#### 2.2.1. Background Variables: Sociodemographic Characteristics, Lifestyle, and Chronic Diseases

We assessed the following sociodemographic characteristics: sex, age, marital status, ethnicity, highest level of education, and net monthly household income. Lifestyle was assessed by asking “Overall, how healthy would you say your lifestyle is?” using five response categories (very healthy, healthy, not healthy/not unhealthy, unhealthy, and very unhealthy). A previous study showed an expected negative correlation between an unhealthy lifestyle (self-evaluation in one question) and independent measures of lifestyle, such as eating fruits and vegetables, and an expected positive correlation between the unhealthy lifestyle question and smoking [36].

Seven self-reported chronic diseases, most frequently found in the older Dutch population, were examined: chronic obstructive pulmonary disease, cardiac disease, diabetes mellitus, cerebrovascular accidents, peripheral arterial disease, cancer, and rheumatoid arthritis or osteoarthritis [30]. Agreement between respondent's self-reported data and data from the general practitioner has been shown to be satisfactory to good for most chronic diseases studied [37]. The total number of self-reported chronic diseases was used for analysis.

#### 2.2.2. Physical Indicators of Frailty

Body Mass Index (BMI) was assessed by self-report, and the other physical frailty indicators were assessed with the Timed Up and Go (TUG) test [38], the LASA Physical Activity Questionnaire (LAPAQ) [39], a hand grip strength test (Martin Vigorimeter; Elmed Inc., Addison, USA), the Shortened Fatigue Questionnaire (SFQ) [40], and the Four-test balance scale [41] (see Table 1).

#### 2.2.3. Disability

Disability was assessed by the Groningen Activity Restriction Scale (GARS), a self-report measure. The GARS is a nondisease-specific instrument to measure disability in activities of daily living (ADL) and instrumental activities of daily living (IADL). Studies have shown that the GARS is an easy to administer, reliable, and valid measure for assessing disability [42, 43]. The GARS comprises 11 items for ADL and 7 for IADL, with response categories: (1) able to perform the activity without any difficulty; (2) able to perform the activity with some difficulty; (3) able to perform the activity with great difficulty; (4) unable to perform the activity independently. The GARS scores (disability total score) range from 18 (no disability) to 72 (maximum disability) and ADL from 11 to 44 and IADL from 7 to 28. The reliability (Cronbach's alpha) of the GARS at baseline was 0.88 in the present study; the reliability was 0.83 and 0.81 for the ADL and IADL subscale, respectively.

### 2.3. Analysis Strategies

After determining the characteristics of participants using descriptive statistics, variables were coded for analysis. The models incorporated linear effects of age, education, income, lifestyle, diseases, and dummy variables “cohabit” (“1” married or cohabiting and “0” rest) and sex (“1” woman and “0” man). The background characteristic “ethnicity” was excluded from further analyses because of the low number of non-Dutch participants (3.7% at baseline).

Bivariate associations between each background variable and physical frailty indicator on the one hand, and disability, ADL disability, and IADL disability on the other were tested using regression analyses. Sequential regression analyses were run to verify which physical frailty indicator improved the prediction of disability after controlling for previous disability, the effects of the background variables, and the remaining indicators. The sequential analyses consisted of three blocks. The effect of previous disability (disability at baseline, assessed in 2007/2008) was estimated in the first block; the results of the first block are equivalent to those of bivariate regression analysis. The second block contained the control variables assessed in 2007/2008 in addition to previous disability. Finally, in the third block, the six physical indicators of frailty were added to the variables of the second block. We tested whether each block increased the prediction of each adverse outcome one year later (2008/2009), using the change in *R* ^2^.

Power analysis using G∗Power 3.1 [44] showed that the regression analysis had a power of 0.8 to detect a small to medium effect size of *f* ^2^ = 0.056 of the block with six frailty indicators on disability one year later, with sample size 250. The power to detect a medium effect size (*f* ^2^ = 0.15) was 0.999. All statistical analyses were conducted using SPSS 18.0 (SPSS, IBM Corp., Somers, NY, United States of America).

## 3. Results

### 3.1. Participant Characteristics

Of the 505 participants at baseline, 14 gave inconsistent responses to basic variables like sex and education at the two time points. Because of these inconsistencies we decided to remove these 14 participants from all analyses and analyzed the data of the remaining 491 participants.

In 2007/2008, the mean age of all participants was 80.0 years and 54.4% was female; 35.4% and 37.6% were widowed in 2007/2008 and 2008/2009, respectively.  Table 2 presents the descriptive statistics of the 491 participants. Column 2 and column 3 show the baseline characteristics of the dropouts (*N* = 241) and the participants assessed twice (*N* = 250), respectively. The average self-reported BMI of participants at baseline who did not drop out was 26.5, with only 3 participants having a BMI smaller than 18.5 (underweight) and 156 having a BMI of 25 or higher (overweight).

A comparison of the dropouts and those who did not drop out on the continuous variables in Table 2 (comparing columns 2 and 3) using a two-sample *t*-test for independent samples showed that those who dropped out were significantly older, scored higher on total, ADL, and IADL disability, and scored worse on physical activity, balance, and hand grip strength (two-tailed test, significance level of 0.05). Although the dropouts differed from those who did not drop out on six variables, these effects were small, and these two groups did not differ on the other twelve variables in Table 2.

### 3.2. Correlations between Frailty Indicators and Regression Analyses

Since previous research suggests that the association between BMI and disability may be nonlinear [45], we tested first if adding a quadratic effect of BMI improved the prediction of a model only including a linear effect of BMI. Since the quadratic effect did not improve the prediction of disability by BMI (increased explained variances of 0.011 for ADL and 0.009 for IADL disability, *P* > 0.05), we decided to only incorporate a linear effect of BMI in further analyses.

The Pearson correlations (not shown in tables) between the physical frailty indicators at baseline were weak to medium. The largest association of −0.34 was between “Up and Go test” and “hand grip strength.” Since none of the associations between the physical frailty indicators was strong, independent effects of some indicators on disability in the multiple regression analyses may be expected.

Table 3 presents the results of the bivariate regression analyses on disability, ADL, and IADL disability, with *P* values significant at 0.05 printed in bold. Not surprisingly, previous disability was a good predictor of disability one year later, with explained variances of 0.65 and 0.49 for ADL and IADL disability, respectively, and 0.65 for disability. Of the control variables, chronic diseases, old age, and lower income were associated with higher scores on all three disability variables; no other control variable was associated with all three disability variables. All six physical frailty indicators were associated with all three disability variables assessed one year later. Effect sizes were mostly medium (*r* = 0.3) to large (*r* = 0.5), or larger [46]. Associations with gait speed were strongest, with Pearson correlations (not shown in Table 3) of 0.70, 0.72, and 0.58 with disability, ADL, and IADL disability, respectively.

Table 3 also summarizes the results of the sequential regression analyses. Although some predictors were rather strongly correlated, there were no multicollinearity problems in the regression analyses, as indicated by a maximum variance inflation factor (VIF) of 2.29 for predicting ADL and 1.83 for predicting IADL disability. The lines “*R* ^2^” indicate how much of the variance of disability, ADL disability, and IADL disability was explained by all predictors together (last row), or in each block (last row of each block), and whether the (increase in) explained variance (Δ*R* ^2^) was statistically significant. The last line of the second block shows that the control variables did not improve the prediction of disability and ADL disability and slightly improved the prediction of IADL disability (increased explained variance of 0.044), after controlling for previous disability. However, none of the individual control variables contributed significantly to the prediction of the disability variables in the final model. The penultimate row reveals that the block of physical frailty indicators increased the predictions significantly of each disability variable one year later (*P* < 0.001), after controlling for the effects of previous disability and the control variables. The indicators explained an additional 6.6%, 8.4%, and 5.8% of disability, ADL, and IADL disability, respectively. Total explained variances were 74% (disability), 75% (ADL disability), and 59% (IADL disability). Of the individual physical frailty indicators, only gait speed significantly improved the prediction of disability, ADL disability, and IADL disability one year later after controlling for the effects of all other variables; fatigue significantly improved the prediction of disability and IADL disability, but not ADL disability. No other individual physical frailty indicator had an effect on any of the disability variables after controlling for the other variables. The effect size of gait speed was medium to large on total (*f* ^2^ = 0.18) and ADL disability (*f* ^2^ = 0.23) and small to medium on IADL disability (*f* ^2^ = 0.084). The effect size of fatigue on all disability measures was small (*f* ^2^ from 0.016 to 0.025).

## 4. Discussion

Disability is a well-known major adverse outcome of physical frailty [11, 12], with frailty being considered as a predisability state [13]. Disability, mostly referring to experienced difficulty in activities of daily living (ADL) and/or difficulty in instrumental activities of daily living (IADL), increases health care utilization and is associated with premature death. In this study we determined which of six individual physical indicators of frailty predict disability, ADL disability, and IADL disability in a representative sample of community-dwelling older persons aged 75 years and older, living in a Dutch city. Our study distinguishes itself from other studies on the long-term association between physical frailty indicators and disability in using a short time span of only one year [25, 47, 48], and using some other frailty indicators than the phenotype of frailty, which was used in, for example, the Cardiovascular Health Study [11] and the Montreal Unmet Needs Study [17]. In addition, the current study differs from our previous studies in which we used the Tilburg Frailty Indicator (TFI), a self-report questionnaire, for measuring frailty [16, 35, 49].

The bivariate regression analyses showed that all six physical indicators of frailty were associated with disability, ADL, and IADL disability. The multiple regression analyses demonstrated that, after controlling for previous disability and other predictors, only gait speed (assessed with the TUG test) was predictive for disability, ADL, and IADL disability, whereas fatigue (assessed with the SFQ) was predictive for disability and IADL disability, but not ADL disability. Although ADL disability represents a more severe form of disability [17], the current study showed similar findings regarding the prediction of ADL and IADL disability by the six physical indicators of frailty. None of the background characteristics consistently predicted disability, after controlling for other variables.

A very high proportion (59% to 75%) of the variance could be explained of disability one year later. This is because baseline disability was one of the predictors. Most important is that even after controlling for baseline disability, the prediction of disability is improved by gait speed and fatigue. That is, even when knowing one's current disability, prediction of his/her future disability can be improved by assessing his/her current gait speed and fatigue. The improvement of the prediction was medium to large for gait speed and small for fatigue; hence, prediction is improved most with gait speed.

The finding that gait speed is associated with disability is supported by several studies [47, 50, 51]. In the present study gait speed was assessed with the TUG test. The TUG test, however, assesses more than gait speed. It requires a transfer from sitting to standing and vice versa, walking and turning, and so the result of the test is not only influenced by gait speed, but strength and balance as well [38, 52]. Then, again, a previous study has shown that the TUG test and gait speed similarly predict ADL disability, falls, and decline in global health in older persons [53]. Moreover, a recent systematic review, based on twelve studies, concluded that slow gait speed is one of the most powerful predictors of ADL disability [28]. These findings suggest that particularly gait speed is responsible for the TUG test's success in predicting future disability.

Several previous studies also showed that fatigue is a predictor of disability [54–56]. Conceptually, fatigue is similar to poor endurance (as indicated by self-report of exhaustion), one of the criteria of the phenotype of frailty [11]. A previous study concluded that endurance is not a significant predictor of ADL disability in older persons [47]. However, it should be noted that the operationalization of endurance varies across studies [17]. We used the SFQ, whereas poor endurance was originally assessed with two questions from the Center for Epidemiologic Studies Depression Scale (CES-D) (“Everything I do is an effort” and “I cannot get going”) [57].

Because only the TUG test and the SFQ predict future disability after controlling for current disability, we recommend that health care workers use these instruments to identify older persons at high risk of disability, ADL, and/or IADL disability. For health care workers it is important to take the complaints of older persons about their gait speed and fatigue seriously, as these persons are at higher risk of becoming ADL and/or IADL disabled and consequently dependent on the help of others.

Our study has several limitations. First, we did not include dementia as one of the chronic diseases. Although we did not exclude older persons with dementia in our study, our measurement of cognitive functioning using the Mini Mental State Examination (MMSE) [58] suggests that persons having memory problems were underrepresented in our study; 3.8% of the participants in 2008/2009 scored <24 on the MMSE (at baseline), which is lower than in another Dutch study among community-dwelling older people (11.9%) [30]. Second, the response rate was not very high on baseline (35.1%) and at follow-up (52.3%), probably because the questions and physical performance measures placed a heavy burden on persons aged 75 and older. A probable consequence of the high nonresponse is an underestimation of the number of frail community-dwelling persons; many older people might have dropped out because they were admitted to a nursing home or had died. Note, however, that differences between dropouts after baseline and those who did not drop out were small and only on six out of eighteen variables assessed. Third, in this study we examined the predictive value of six physical indicators of frailty in a one-year period. Perhaps the predictive value of these indicators of frailty differs when a follow-up of two years or more is used. Research showed that the onset of disability was predicted by physical activity after a follow-up of three years [59] and ten years [21] and hand grip strength after a follow-up of nine years [25]. Fourth, it is important to consider the effects of potential overlap between the operationalization of the concepts frailty and disability. For instance, gait speed was the strongest predictor of ADL, and ADL includes three items referring to walking. However, it should be noted that gait speed predicts future ADL, even after controlling for current ADL, which contains the same three walking items. Thus apparently gait speed still contains aspects relevant to the prediction of ADL, perhaps due to the fact that it is not a self-reported measure like ADL. The strength of this study is that the analyses included well-validated measures of independent variables Body Mass Index, gait speed, physical activity, fatigue, hand grip strength, balance, and dependent variable disability.

Several intervention studies aiming at the prevention of disability in older persons using physical indicators of frailty as inclusion criteria have been reported; many of these studies focused on physical exercise interventions [60, 61]. High-intensity multicomponent exercise interventions, addressing a variety of physical indicators of frailty, seem to have a positive effect on ADL and IADL disability [60]. The findings of our study showed that these interventions should be focused at least on improving gait speed and fatigue of frail older persons. An investment in the further development of interventions preventing or delaying disability seems necessary.

## Figures and Tables

**Table 1:** Measures used to operationalize the physical frailty indicators.

Physical frailty indicator	Instrument	Operationalization
Body Mass Index (BMI)	Self-report	BMI was calculated by dividing weight in kilograms by height in meters squared.

Gait speed	Timed Up and Go (TUG) test	The TUG test measures the time the respondent takes to rise from an armchair, walk three meters, and return to the chair.

Physical activity	LASA Physical Activity Questionnaire (LAPAQ), self-report	Participants were asked how often and how long in the 2 weeks before the interview they had walked, bicycled, and performed sport activities and light and heavy household activities. The total time spent on physical activity was calculated by multiplying the frequency by the duration of each activity, divided by 14.

Hand grip strength	Martin Vigorimeter	Hand grip strength of the dominant hand was measured three times. The highest value was used.

Fatigue	Shortened Fatigue Questionnaire (SFQ), self-report	The SFQ consists of four statements which the person answers by checking an item at a 7-point scale. In this study a 3-point scale was used (yes that is correct, that is more or less correct, and no that is not correct).

Balance	Four-test balance scale	The Four-test balance scale includes four timed static balance tasks of increasing difficulty that are completed without assistive devices. Respondents were asked to hold each position for 10 seconds. In this study participants performed three tasks: side-by-side, semitandem, and tandem. If respondents could not perform at least one of these three tasks, then balance was coded as poor.

**Table 2:** Characteristics of respondents (2007/2008, *N* = 491; 2008/2009, *N* = 250)^1^.

Characteristic	Dropouts (*n* = 241) 2007/2008 *n* (%)	(*n* = 250) 2007/2008 *n* (%)	(*n* = 250) 2008/2009 *n* (%)
Sociodemographic characteristics			
Age, mean ± SD	80.6 ± 4.4	79.4 ± 3.6	80.4 ± 3.6
Sex, % of women	133 (55.2)	134 (53.6)	134 (53.6)
Marital status			
Married or cohabiting	129 (53.5)	137 (54.8)	130 (52.0)
Not married	18 (7.5)	19 (7.6)	19 (7.6)
Divorced	7 (2.9)	7 (2.8)	7 (2.8)
Widowed	87 (36.1)	87 (34.8)	94 (37.6)
Ethnicity			
Dutch	228 (94.6)	243 (98.0)	242 (98.0)
Other	13 (5.4)	5 (2.0)	5 (2.0)
Education			
None or primary	124 (54.4)	115 (47.1)	115 (46.6)
Secondary	71 (31.1)	80 (32.8)	85 (34.4)
Higher	33 (14.5)	49 (20.1)	47 (19.0)
Missing values (*N*)	13	6	3
Monthly income*			
€600 or less	2 (1.0)	1 (0.4)	3 (1.4)
€601–€900	9 (4.6)	10 (4.4)	10 (4.5)
€901–€1200	51 (26.2)	45 (19.8)	46 (20.9)
€1201–€1500	44 (22.6)	59 (26.0)	50 (22.7)
€1501–€1800	35 (17.9)	32 (14.1)	33 (15.0)
€1801–€2100	24 (12.3)	25 (11.0)	28 (12.7)
€2101 or more	30 (15.4)	55 (24.2)	50 (22.7)
Missing values (*N*)	46	23	30
Self-perceived lifestyle			
Very healthy	27 (11.2)	35 (14.1)	47 (19.0)
Healthy	186 (77.2)	195 (78.3)	184 (74.5)
Not healthy, not unhealthy	19 (7.9)	13 (5.2)	10 (4.0)
Unhealthy	9 (3.7)	6 (2.4)	5 (2.0)
Very unhealthy	0 (0.0)	0 (0.0)	1 (0.4)
Chronic diseases, mean ± SD	1.4 ± 1.2	1.3 ± 1.1	1.4 ± 1.1
COPD	38 (15.8)	37 (14.8)	40 (16.0)
Cardiac disease	39 (16.2)	50 (20.0)	53 (21.2)
Diabetes mellitus	48 (19.9)	44 (17.6)	51 (20.4)
Cerebrovascular accidents	15 (6.2)	13 (5.2)	13 (5.2)
Peripheral arterial disease	40 (16.6)	33 (13.2)	34 (13.6)
Cancer	22 (9.1)	18 (7.2)	22 (8.8)
RA or osteoarthritis	138 (57.3)	138 (55.2)	149 (59.6)
Physical indicators of frailty			
Body Mass Index, kg/m^2^, mean ± SD	26.0 ± 3.9	26.5 ± 4.1	26.5 ± 4.0
Gait speed, sec, mean ± SD	15.1 ± 9.1	13.7 ± 6.6	13.7 ± 8.7
Physical activity, min/day, mean ± SD	100.5 ± 78.7	116.0 ± 84.0	133.0 ± 95.0
Hand grip strength, PKa, mean ± SD	69.3 ± 21.4	73.1 ± 19.4	66.6 ± 20.8
Fatigue, mean ± SD	6.6 ± 2.7	6.4 ± 2.6	6.3 ± 2.6
Poor balance	125 (53.9)	107 (44.6)	106 (42.4)
Disability	28.7 ± 9.2	26.2 ± 8.8	27.9 ± 9.9
ADL disability	15.3 ± 4.7	14.4 ± 4.7	15.3 ± 5.2
IADL disability	13.4 ± 5.4	11.8 ± 4.9	12.5 ± 5.5
MMSE, mean ± SD	27.0 ± 3.6	28.0 ± 2.1	27.8 ± 2.3
<24 (range 0–30)	29 (12.2)	10 (4.0)	10 (4.1)

Sec: seconds; PKa: Kilopascal; SD: standard deviation; COPD: chronic obstructive pulmonary disease; RA: rheumatoid arthritis; MMSE: Mini Mental State Examination.

*46 cases were missing (dropouts 2007/2008); 23 cases were missing (2007/2008); 30 cases were missing (2008/2009).

^1^There were occasional missing values on many variables. Only for education and income was the number of missing values larger than ten (>4%) on at least one occasion. Only for these variables we included a count of the number of missing values in the table.

**Table 3:** Effect of previous disability, background characteristics, and physical indicators of frailty on disability, ADL disability, and IADL disability: results of bivariate and sequential linear regression analysis.

	Disability						ADL						IADL					
	Bivariate			Multiple			Bivariate			Multiple			Bivariate			Multiple		
	*B*	se	*P*	*B*	se	*P*	*B*	se	*P*	*B*	se	*P*	*B*	se	*P*	*B*	se	*P*
Block 1																		
Previous disability	0.952	0.039	**<0.001**	0.598	0.068	**<0.001**	0.929	0.037	**<0.001**	0.593	0.064	**<0.001**	0.856	0.048	**<0.001**	0.525	0.073	**<0.001**
*R* ^2^				0.651		**<0.001**				0.654		**<0.001**				0.492		**<0.001**
Block 2																		
Sex (women)	2.503	1.249	**0.046**	0.100	0.837	0.906	1.889	0.638	**0.003**	0.112	0.418	0.790	0.575	0.698	0.411	−0.139	0.594	0.816
Age	0.410	0.173	**0.019**	0.013	0.104	0.904	0.178	0.089	**0.048**	−0.028	0.052	0.595	0.223	0.096	**0.021**	0.033	0.073	0.651
Marital status (cohabit)	−1.580	1.258	0.210	1.042	0.813	0.201	−1.317	0.645	**0.042**	0.358	0.412	0.386	−0.218	0.701	0.756	0.712	0.572	0.215
Education	−0.562	0.263	**0.034**	−0.084	0.163	0.605	−0.280	0.136	**0.041**	−0.003	0.082	0.970	−0.274	0.146	0.063	−0.083	0.115	0.467
Income	−1.138	0.415	**0.007**	−0.250	0.269	0.353	−0.575	0.215	**0.008**	−0.080	0.136	0.557	−0.563	0.231	**0.016**	−0.167	0.188	0.377
Self-perceived lifestyle	3.495	1.149	**0.003**	−0.016	0.698	0.982	1.121	0.602	0.063	−0.563	0.351	0.111	2.371	0.630	**<0.001**	0.583	0.491	0.237
Chronic diseases	3.053	0.563	**<0.001**	0.397	0.376	0.293	1.459	0.293	**<0.001**	0.143	0.190	0.453	1.581	0.315	**<0.001**	0.281	0.263	0.287
Δ*R* ^2^				0.021		0.100				0.014		0.337				0.044		**0.014**
Block 3																		
Body Mass Index	0.662	0.150	**<0.001**	0.004	0.107	0.974	0.349	0.077	**<0.001**	0.014	0.054	0.803	0.312	0.084	**<0.001**	0.001	0.074	0.984
Gait speed	0.972	0.064	**<0.001**	0.395	0.068	**<0.001**	0.511	0.031	**<0.001**	0.232	0.035	**<0.001**	0.458	0.042	**<0.001**	0.177	0.045	**<0.001**
Physical activity	−0.040	0.007	**<0.001**	−0.005	0.005	0.264	−0.016	0.003	**<0.001**	−0.001	0.002	0.640	−0.024	0.004	**<0.001**	−0.004	0.003	0.175
Hand grip strength	−0.228	0.029	**<0.001**	−0.025	0.023	0.272	−0.125	0.015	**<0.001**	−0.020	0.011	0.083	−0.102	0.017	**<0.001**	−0.007	0.016	0.666
Fatigue	1.799	0.210	**<0.001**	0.357	0.173	**0.040**	0.855	0.111	**<0.001**	0.144	0.086	0.094	0.947	0.118	**<0.001**	0.249	0.119	**0.038**
Balance	8.456	1.171	**<0.001**	0.789	0.794	0.322	4.201	0.611	**<0.001**	0.728	0.393	0.066	4.273	0.662	**<0.001**	0.191	0.556	0.731
Δ*R* ^2^				0.066		**<0.001**				0.084		**<0.001**				0.058		**<0.001**

*R* ^2^ total				0.738		**<0.001**				0.752		**<0.001**				0.594		**<0.001**

Notes: *B*, se, and *P* are unstandardized regression coefficient, its standard error, and the two-tailed *P* value of the *t*-test of *B*. *R* ^2^ is the proportion of explained variance of the regression model; Δ*R* ^2^ is the change in *R* ^2^ after adding the predictors of one block to the model.
